# The Coventry Programme

**Published:** 1921-01-29

**Authors:** 


### The Coventry Programme.

### SIR JOHN BARNSLEY'S IDEAS.

Brigadier-General Sir John Barnsley was the principal speaker at the annual meeting of the Coventry and Warwickshire Hospital, under the presidency of the Mayor, Councillor W. H. Grant. Sir John Barnsley said that he found from the report that in twenty years the income went up from £3,641 to £28,978, while the cost increased from £4,102 to £30,385 last year. Although the institution hardly paid its way they came very near to doing it, and that was a source of great satisfaction.

This success was largely due to the contribution of the Hospital Saturday collections, which increased by £6,650 over the previous year, while the increase in subscriptions from employers amounted to £1,570, largely because of an appeal made by the Mayor. He paid a high tribute to those engaged in the Hospital Saturday collection in raising the magnificent sum of £15,000 last year. His experience is that if the case of the hospitals is put before the workers in the great cities properly they are not appealed to in vain. (Applause.) They, however, want to be assured that other classes of the community are doing their share. (Hear, hear.) Therefore he puts propaganda in the first place of their programme as hospital workers, and he is sure the public generally would respond.

The public is becoming sick and tired of spasmodic appeals, and it would be far better to rely upon subscriptions from all classes properly organised. He regrets that the insurance companies have never recognised the claims of the hospitals upon them. The hospitals are looking to the future with great anxiety, owing to the heavy calls upon the country and the increasing taxes and rates. It is suggested in various quarters that the hospitals should become State institutions, but there are many pertinent points to be considered before the voluntary system is given up, especially as a great authority upon hospital management has stated it would increase the cost three times.

Recent experience of State control has not led them to suppose there would be any advantage by the State taking over the hospitals. He did not say there were not ways in which the State or municipal corporations could help. There are Poor-law infirmaries which are not fully used, and he suggests these unused places might be handed over to the hospitals to be used as part of their voluntary work, and grants in aid provided for the necessary accommodation and administration. It would be a great shame to damp that great stream of human charity which flows to all their hospitals.

